# Mutations in *CRBN* and other cereblon pathway genes are infrequently associated with acquired resistance to immunomodulatory drugs

**DOI:** 10.1038/s41375-021-01373-4

**Published:** 2021-08-09

**Authors:** J. R. Jones, A. Barber, Y-V Le Bihan, N. Weinhold, C. Ashby, B. A. Walker, C. P. Wardell, H. Wang, M. F. Kaiser, G. H. Jackson, F. E. Davies, R. Chopra, G. J. Morgan, C. Pawlyn

**Affiliations:** 1 The Institute of Cancer Research, London, UK; 2 Brighton and Sussex Medical School, Brighton, UK; 3 Kings College Hospital NHS Foundation Trust, London, UK; 4 Department of Internal Medicine V, University Hospital of Heidelberg, Heidelberg, Germany; 5 Department of Biomedical Informatics, University of Arkansas for Medical Sciences, Arkansas, USA; 6 Indiana University School of Medicine, Indiana, USA; 7 The Royal Marsden Hospital, London, UK; 8 Northern Institute for Cancer Research, Newcastle University, Newcastle upon Tyne, UK; 9 Perlmutter Cancer Center, NYU Langone Health, New York, USA; 10 Apple Tree Partners, London, UK

**Keywords:** Myeloma, Myeloma

Immunomodulatory drugs (IMiDs) are the current backbone of standard and experimental combination myeloma therapies at all stages of the disease. However, the majority of patients inevitably relapse and the mechanisms of resistance are still poorly understood. Previous studies looking for genetic drivers of resistance have looked for changes in core members of the CRL4^CRBN^ E3-ubiquitin ligase complex (CUL4-RBX1-DDB1-CRBN) and identified mutations in the protein to which IMiDs bind, cereblon (CRBN), but at a rate that cannot account for resistance in the majority of patients [1, 2]. Several in vitro studies have now identified novel upstream and downstream regulators of CRBN activity that could have a role in IMiD resistance and have not been previously examined in patient samples [3–6]. In this study paired presentation and relapse samples from newly diagnosed patients recruited to a clinical trial of IMiD therapies were used to investigate the role of mutations in all genes currently implicated in IMiD activity. Infrequent mutations in CRBN itself were identified that could be a cause of IMiD resistance in some patients. CRBN and other genes in the IMiD response pathway were mutated at low frequency and, in many cases, at low clonal fraction suggesting that mechanisms other than mutations, for example post-translational modification or epigenetic alterations, may underlie resistance acquisition.

The mechanism of action of IMiDs in myeloma has been partly elucidated. Binding of the IMiDs within a tri-tryptophan pocket on the surface of CRBN alters substrate specificity of the CRL4^CRBN^ E3-ubiquitin ligase complex, leading to the ubiquitination of neosubstrates such as the C2H2 zinc finger domain-containing B-cell transcription factors Ikaros (gene name: *IKZF1*) and Aiolos (*IKZF3*), which are then degraded *via* the proteasome [7–9]. Ikaros and Aiolos degradation results in the subsequent downregulation of their target genes, including interferon regulatory factor 4 (IRF4) and c-Myc, which are transcription factors regulating stages of B-cell development. IRF4 is thought to be the key driver of aberrant transcriptional regulation in myeloma, with its downregulation causing cell death. Clinical response rates to IMiDs are high but the majority of patients will inevitably relapse. Understanding IMiD resistant and refractory states is therefore imperative to help us improve patient outcomes.

Recent in vitro studies have improved our understanding of the mechanisms of control of the cereblon IMiD response pathway implicating genes encoding components of the core CRL4^CRBN^ E3-ligase complex and the COP9 signalosome, as well as IMiD-induced neosubstrates and downstream targets [3–6, 10]. Liu et al. [4], Sievers et al. [5], and Tateno et al. [6] performed genome-wide screens of myeloma cell lines to identify genes implicated in IMiD response. All three screens identified subunits of the CRL4^CRBN^ complex as important for IMiD sensitivity, in line with the previously published IMiD mechanism of action [11]. The screens also all identified components of the COP9 signalosome as important in determining IMiD sensitivity, including *COPS1* [10], *COPS2,* and *COPS4* [4, 6] and *COPS5* [3–6, 10]. Tateno et al. [6], in addition, identified *NEDD8*, which is critical for the activation of the CRL4 E3-ligase, as having a role in IMiD response and highlighted the influence of the subcellular localisation of CRBN. Sievers et al. [5] found that the loss of E2 ubiquitin-conjugating enzymes *UBE2D3* and *UBE2G1*, as well as *DEPDC5*, a GAP Activity Towards Rags complex 1 (GATOR1) member, reduced degradation of the neosubstrate IKZF3. Zinc finger transcription factors such as IKZF1, IKZF3, and SALL4 are well-described neosubstrates for CRBN in the presence of the IMiDs thalidomide, lenalidomide, and pomalidomide. Donovan et al. [3], using a proteomic screen, demonstrated the degradation of these neosubstrates upon IMiD treatment of cell lines and also identify a number of other neosubstrates, many of which are zinc finger proteins; ZNF827, ZNF98, and GZF1. They also identify non-zinc finger targets CSNK1A1 and DTWD1 [3].

Together these recent studies give a clearer understanding of the control of CRL4^CRBN^ activity via neddylation and deneddylation (Fig. 1A) and of CRL4^CRBN^ neosubstrates. In this study we applied this knowledge to investigate the role of mutations in these genes, as well as those of the core CRL4^CRBN^ E3-ubiquitin ligase complex, in the acquisition of resistance to lenalidomide, the most widely clinically used IMiD. We have previously reported a study examining the impact of maintenance lenalidomide and depth of response on the genetics and sub-clonal structure of relapsed disease. Study samples were selected from newly diagnosed patients enroled in the UK National Cancer Research Institute Myeloma XI trial (NCT01554852) [12, 13] for whom adequate DNA volumes were available at the time of study design. 56 patients who received immunomodulatory drug induction therapy followed by either lenalidomide maintenance (*n* = 30) or observation (*n* = 26), and subsequently relapsed, were selected. Whole exome sequencing analysis, median depth 122x for tumour samples and 58x for paired germline controls, had been performed as previously described [13] and summarised in Supplementary Methods.

**Fig. 1:**
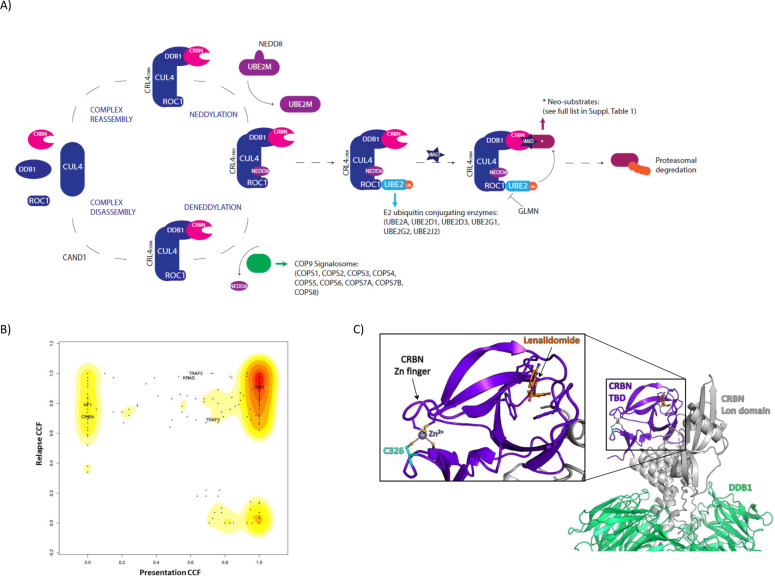
CRL4^CRBN^ E3-ubiquitin ligase complex regulation and features of the CRBN mutation identified. **A** Diagram outlining the regulation of the CRL4^CRBN^ E3-ubiquitin ligase complex, indicating the “CRBN/IMiD” genes analysed in this study. **B** The clonal evolution pathway from presentation to relapse determined by the Cancer Clonal Fraction for all mutations in the patient sample with a *CRBN* mutation detected by whole-exome sequencing using 2D Kernal Density Estimate plot of the CCF. This suggests a branching evolutionary pattern leading to relapse with distinct clusters of mutations seen at presentation only, relapse only, and at both time points. **C** Structure of Lenalidomide-bound human CRBN/DDB1 complex, highlighting the participation of Cys326 in a Zinc finger motif within the Thalidomide Binding Domain (TBD) of CRBN. Figure generated with Pymol using coordinates from PDB 4TZ4 (10.1038/nsmb.2874).

From recent publications, a list of 42 genes involved in cereblon pathway regulation and IMiD response was curated, termed “CRBN/IMiD genes” (Fig. 1A and Supplementary Table 1). The frequency of non-synonymous mutations and deletions in CRBN/IMiD genes in the patient dataset was examined and the cancer clonal fraction (CCF) between the diagnosis and relapse samples was compared. 12/42 (29%) of these genes were found to be mutated in the dataset with a total of 17 mutations identified. With the exception of *SALL4*, which was mutated in three patients, no other CRBN/IMiD gene was mutated in more than two patients.

14/56 (25%) of patients had a mutation in a CRBN/IMiD gene either at presentation, relapse, or at both time points (Table 1). Three patients had mutations in two different genes. 6/14 of the patients with CRBN/IMiD mutations (43%) had received lenalidomide maintenance and 8/14 (57%) had been in the observation arm of the trial. Of the 17 mutations, 9 (53%) arose in patients who had received lenalidomide maintenance and 8 (47%) in patients who were observed. Importantly, in the patients receiving lenalidomide maintenance, 6 of the 9 (67%) mutations had a higher cancer clonal fraction (CCF) in the relapse sample, suggesting they may have been selected for by exposure to treatment. Two of these mutations were only detected at relapse and not at presentation, in *CRBN* (CCF 0.71 at relapse) and *FAM83F* (CCF 0.54 at relapse). Comparatively, in mutations identified in patients undergoing observation, only 3 of the 8 (38%) mutations had a higher CCF at relapse compared with the presentation. The only deletion in any of the CRBN/IMiD genes was in *SETX* in one patient at relapse.

**Table 1:** Mutations identified in CRBN/IMiD genes in patient samples.

Putative function/ complex	Genes	Ref.	Patient no.	Mutation	Mutation type	Amino acid change	Maintenance allocation	CCF at diagnosis	CCF at relapse
**Core E3-ligase complex**	***CRBN***	4,5,6	63	3:3195148A > C	Missense variant	Cys326Gly	Lenalidomide	0	0.71
	***DDB1***	4,5,6	27	11:61070633A > C	Splice region variant	N/A	Observation	0.99	0.69
			61	11:61089144T > G	Missense variant	Lys383Thr	Observation	0	0.21
**E2 ubiquitin conjugating enzymes**	***UBE2G2***	3	106	21:46199134G > A	Splice region variant	N/A	Lenalidomide	0.89	1.00
	***UBE2J2***	3	1	1:1203352C > G	Missense variant	Lys7Asn	Lenalidomide	0.15	0
			53	1:1190726C > T	Missense variant	Val229Ile	Observation	0	0.18
**Splicing**	***SETX***	6	63	9:135205643C > T	Missense variant	Asp448Asn	Lenalidomide	1.0	0
			85	9:135203409A > C	Missense variant	Asp1192Glu	Lenalidomide	0.06	0.15
	***SLU7***	6	26	5:159842258G > A	Missense variant	Ser15Leu	Lenalidomide	0.39	0.67
**IMiD neo-substrates**	***DTWD1***	3	77	15:49935593G > C	Missense variant	Asp245His	Observation	1.00	0.9
	***FAM83F***	3	26	22:40417571G > A	Missense variant	Gly353Ser	Lenalidomide	0	0.54
	**GZF1**	3	43	20:23345844A > C	Missense variant	Gln275Pro	Observation	0.22	0.05
	***SALL4***	3	1	20:50401117G > A	Missense variant	Ser950Leu	Lenalidomide	0.23	0
			39	20:50408653C > A	Missense variant	Gln123His	Lenalidomide	0.49	0.52
			95	20:50408538G > A	Missense variant	Pro162Ser	Observation	1.00	0.24
	***ZFP91***	3	28	11:58378474G > C	Missense variant	Glu223Asp	Observation	0.93	1.00
	***ZNF98***	3	75	19:22575634G > T	Missense variant	His135Asn	Observation	0.95	0.95

CCF cancer clonal fraction.

A single patient in the study had a *CRBN* mutation identified only at relapse at g.3:3195148A > C, encoding a Cys326Gly sequence modification at the protein level. The absence of the CRBN mutation at presentation was confirmed by deep sequencing (769 reference reads and 0 alternative reads). Clonal analysis showed that the CRBN mutation arose as a late event in a new subclone supporting the hypothesis that it contributed to the acquisition of lenalidomide resistance and clinical relapse (Fig. 1B and Supplementary Fig. 1). This patient received thalidomide-based induction (cyclophosphamide, thalidomide, and dexamethasone) followed by 9 cycles of lenalidomide maintenance and achieved a minimal residual disease negative (MRD-ve) complete response prior to relapse. Interestingly, Cys326 is one of 4 cysteines in CRBN coordinating a single Zinc ion to form a Zn finger motif, which stabilises the Thalidomide Binding Domain (TBD) of the protein (Fig. 1C). Zn fingers are a common motif found in many types of proteins and their protein-stabilising properties are well established. Mutations in Zn fingers have been associated with several types of diseases, including cancer (e.g. mutations on MDM2 or on tumour suppressor ZNF750). In the case of CRBN, an in vitro site-directed mutagenesis study on CRBN TBD has shown that mutation of any of the 4 cysteines involved in the Zn finger formation led to protein misfolding and aggregation [14]. Another study has linked a mutation on CRBN Cys394, another cysteine involved in the Zn finger, to an inherited neurodevelopmental disorder, which could be due to the lack of functional CRBN during developmental phases [15]. Finally, the 4 cysteines of CRBN are conserved amongst vertebrates, further indicating it is vital for cereblon function.

Our findings suggest that mutations in CRBN/IMiD pathway genes occur in some myeloma patients exposed to immunomodulatory drugs as part of first line therapy but are not the major mechanism underlying resistance to these drugs. In the majority (~75%) of patients, there were no known CRBN/IMiD pathway genes mutated. In addition to DNA variants, previous studies have suggested that variant transcripts of cereblon, notably exon 10-spliced, may be associated with lenalidomide-resistant disease [2]. Transcriptional changes were not examined in this study in which paired RNA-seq data was not available but given the low rate at which these have been reported there remains a large amount of resistance unaccounted for by any known mechanism. Alternative mechanisms, such as epigenetic modification of target expression or post-translational modification, may be implicated in the acquisition of immunomodulatory drug resistance and subsequent clinical relapse and should be further explored.

## Supplementary information

Supplementary Material

## Data Availability

Original sequencing data files are available via https://ega-archive.org/datasets/EGAD00001004846/.
