# Supplementary material for: Mutations in CRBN and other cereblon pathway genes are infrequently associated with acquired resistance to immunomodulatory drugs
Source: Leukemia. 2021 Aug 9;35(10):3017–20. doi: 10.1038/s41375-021-01373-4 (PMC8478640; doi:10.1038/s41375-021-01373-4)

**Supplementary Methods**

**Whole exome sequencing**

DNA was isolated from patient plasma cells following selection using CD138+ MACSorting (Miltenyi Biotech, Bisley, United Kingdom) from bone marrow aspirate samples. Control DNA was obtained from peripheral blood samples. WES libraries were prepared using the SureSelectQXT sample prep kit and the SureSelect Clinical Research Exome kit (Agilent), with additional baits covering the immunoglobulin and *MYC* loci, as previously described.^1^ Paired-end sequencing was performed to a median sequencing depth of 122x for tumour samples and 58x for controls using a HiSeq2500 (Illumina). All variants were called using MuTect (version 1.1.4). The distribution of mutant alleles determined by the variant allele frequency (VAF) was mapped using the R package SciClone. Cancer clonal fractions (CCF) were calculated for all mutations according to the method of Stephens *et al* and plotted using Kernal density estimation to infer clonal structure at presentation and relapse.^2^ CCF values are quoted as 1.0 when the estimate was >=1.0. PhyloWGS (version 20150714, Github) was used to build phylogenetic evolutionary prediction trees. Each numbered node has a corresponding mutational profile deemed to be in order of occurrence.

Copy number aberrations (CNA) and chromosomal translocations at presentation and relapse were determined in all patients. Copy number was assessed using both multiplexed ligation-dependant probe amplification (MLPA) (SALSA MLPA P425-B1 multiple myeloma probemix, MRC Holland, Amsterdam, The Netherlands) and the bioinformatics assessment tool sequenza (version 2.1.2).^3,4^ Paired MLPA and sequenza data was available for 90/112 (80%) tumour samples, with a consensus between MLPA and sequenza apparent in 85/90 samples (94%). For the five patients where a mismatch was observed sequenza was used to call the copy number profile.

Translocations were determined using MANTA (version 0.29.3).^5^ For 46% (51/112) of patient samples translocations involving the immunoglobulin heavy chain (IGH) were also assessed using multiplexed qRT-PCR.^6^ A consensus between MANTA and qRT-PCR was observed in 84% (43/51). For the 8 patients where a mismatch was seen, the integrative genomics viewer (IGV) was used to confirm or exclude the translocation.

Deletions and mutations of tumour suppressor genes and oncogenes were determined using sequenza, MLPA and MuTect. All suspected mono-allelic and bi-allelic CNA events were confirmed by manual interrogation of BAM files using IGV. Bi-allelic inactivation was also called in patients with evidence of a non-synonymous mutation with mono-allelic loss or a single mutation with a VAF of ≥80%.

**References**

1. Walker BA, Wardell CP, Murison A, et al. APOBEC family mutational signatures are associated with poor prognosis translocations in multiple myeloma. *Nat Commun* 2015; **6**: 6997.

2. Stephens PJ, Tarpey PS, Davies H, et al. The landscape of cancer genes and mutational processes in breast cancer. *Nature* 2012; **486**(7403): 400-4.

3. Boyle EM, Proszek PZ, Kaiser MF, et al. A molecular diagnostic approach able to detect the recurrent genetic prognostic factors typical of presenting myeloma. *Genes Chromosomes Cancer* 2015; **54**(2): 91-8.

4. Favero F, Joshi T, Marquard AM, et al. Sequenza: allele-specific copy number and mutation profiles from tumor sequencing data. *Ann Oncol* 2015; **26**(1): 64-70.

5. Rausch T, Zichner T, Schlattl A, Stutz AM, Benes V, Korbel JO. DELLY: structural variant discovery by integrated paired-end and split-read analysis. *Bioinformatics* 2012; **28**(18): i333-i9.

6. Kaiser MF, Walker BA, Hockley SL, et al. A TC classification-based predictor for multiple myeloma using multiplexed real-time quantitative PCR. *Leukemia* 2013; **27**(8): 1754-7.

**Supplementary Table 1**

| **Putative function/ complex** | **Gene symbol** | **Gene product** | **Ref*.** |
| --- | --- | --- | --- |
| **Core E3-ligase complex** | ***CRBN*** | *Cereblon* | 4,5,6 |
|  | ***DDB1*** | *Damage-specific DNA binding protein 1* | 4,5,6 |
|  | ***ROC1*** | *Regulator of cullins-1, or RING box protein 1 (RBX-1)* | 6 |
|  | ***CUL4A*** | *Cullin 4A* | 6 |
| **Neddylation and deneddylation** | ***CAND1*** | *Cullin Associated And Neddylation Dissociated protein 1* | 5 |
|  | ***COPS1*** | *COP9 signalosome subunit 1* | 5,6 |
|  | ***COPS2*** | *COP9 signalosome subunit 2* | 4,5 |
|  | ***COPS3*** | *COP9 signalosome subunit 3* | 5 |
|  | ***COPS4*** | *COP9 signalosome subunit 4* | 4,5 |
|  | ***COPS5*** | *COP9 signalosome subunit 5* | 4,5,6 |
|  | ***COPS6*** | *COP9 signalosome subunit 6* | 4 |
|  | ***COPS7A*** | *COP9 signalosome subunit 7A* | 4,5 |
|  | ***COPS7B*** | *COP9 signalosome subunit 7B* | 4,5 |
|  | ***COPS8*** | *COP9 signalosome subunit 8* | 4 |
|  | ***GLMN*** | *Glomulin, FKBP Associated Protein* | 5 |
|  | ***NEDD8*** | *Neural Precursor Cell Expressed, Developmentally Down-Regulated 8* | 6 |
|  | ***UBE2M*** | *NEDD8 conjugating enzyme Ubc12* | 5  4 |
| **E2 Ubiquitin conjugating enzymes** | ***UBE2A*** | *Ubiquitin-conjugating enzyme E2A* | 4 |
|  | ***UBE2D1*** | *Ubiquitin-conjugating enzyme E2D 1* | 6 |
|  | ***UBE2D3*** | *Ubiquitin-conjugating enzyme E2D 3* | 5 |
|  | ***UBE2G1*** | *Ubiquitin-conjugating enzyme E2G 1* | 4,5 |
|  | ***UBE2G2*** | *Ubiquitin-conjugating enzyme E2G 2* | 3 |
|  | ***UBE2J2*** | *Ubiquitin-conjugating enzyme E2J 2* | 3 |
| **GATOR complex** | ***DEPDC5*** | *Neural Precursor Cell Expressed, Developmentally Down-Regulated 8* | 5 |
| **Splicing** | ***EIF4A3*** | *Eukaryotic Translation Initiation Factor 4A3* | 6 |
|  | ***PHF5A*** | *PHD Finger Protein 5A* | 6 |
|  | ***SETX*** | *Senataxin* | 6 |
|  | ***SLU7*** | *Pre-MRNA-Splicing Factor SLU7* | 6 |
| **Nuclear import proteins** | ***KPNB1*** | *Karyopherin Subunit Beta 1* | 6 |
| **IMiD neo-substrates** | ***CSNK1A1*** | *Casein Kinase 1 Alpha 1* | 3 |
|  | ***DTWD1*** | *DTW Domain-Containing Protein 1* | 3 |
|  | ***FAM83F*** | *Family With Sequence Similarity 83 Member F* | 3 |
|  | ***GSPT1*** | *G1 To S Phase Transition protein 1* | 3 |
|  | ***GZF1*** | *GDNF Inducible Zinc Finger Protein 1* | 3 |
|  | ***IKZF1*** | *IKAROS family zinc finger 1* | 3 |
|  | ***IKZF3*** | *IKAROS family zinc finger 3* | 3 |
|  | ***SALL4*** | *Spalt Like Transcription Factor 4* | 3 |
|  | ***ZNF653*** | *Zinc finger protein 653* | 3 |
|  | ***ZNF692*** | *Zinc finger protein 692* | 3 |
|  | ***ZNF827*** | *Zinc finger protein 827* | 3 |
|  | ***ZFP91*** | *Zinc finger protein 91* | 3 |
|  | ***ZNF98*** | *Zinc finger protein 98* | 3 |

*Reference numbers refer to those in the main manuscript.

**Supplementary Figure 1**

**Clonal Evolution Pathway**

The clonal evolution pathway from presentation to relapse determined by the Cancer Clonal Fraction for all mutations in the patient sample with a *CRBN* mutation detected by WES using PhyloWGS modelling. This is consistent with the KDE showing that mutations in both *CRBN* and *NF1* occurred in a late branching event. *DIS3*, noted at both presentation and relapse was seen at a very early time point, likely to have occurred following the initiation event t(14;16) and acquisition of del(1p). A *MYC* translocation was also noted as a new event at relapse.


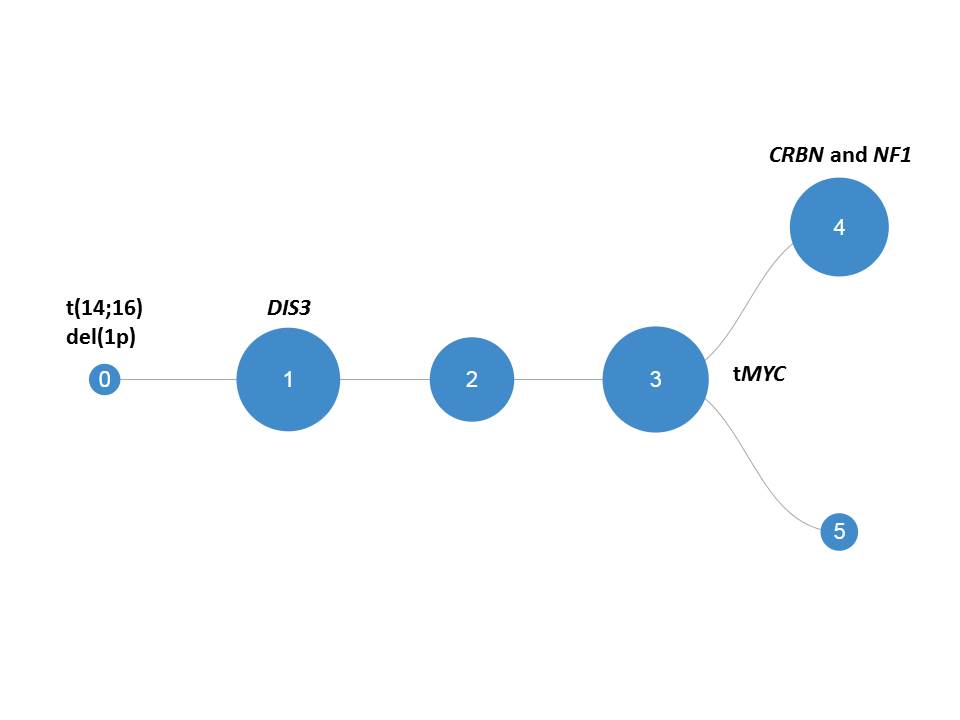

Supplement: Supplementary file 1 — Supplementary Material [file 41375_2021_1373_MOESM1_ESM.docx]
